# Biocatalytic activity of *Aspergillus niger* xylanase in paper pulp biobleaching

**DOI:** 10.1007/s13205-016-0480-0

**Published:** 2016-08-11

**Authors:** A. Sridevi, A. Sandhya, G. Ramanjaneyulu, G. Narasimha, P. Suvarnalatha Devi

**Affiliations:** 1 Department of Applied Microbiology, Sri Padmavathi Mahila Visvavidyalayam, Tirupati, AP India; 2 Department of Microbiology, Sri Krishnadevaraya University, Anantapur, AP India; 3 Applied Microbiology Lab, Department of Virology, Sri Venkateswara University, Tirupati, AP India

**Keywords:** Biobleaching, Effluent characteristics, FTIR, SEM, Xylanase

## Abstract

Xylanase is a hemicellulase enzyme that catalyses the hydrolysis of xylan to xylose which is widely used in processing of feed, pulp and paper. It is produced by many microorganisms especially filamentous fungi like *Trichoderma* and *Aspergillus*. A potential xylanolytic fungal isolate *Aspergillus niger* was isolated from forest soils of Tirumala, AP, India, and its crude enzyme was checked for its potential in paper bleaching. Under submerged fermentation, production of xylanase, cellulase, biomass, total protein and sugar released were analysed after 7 days of incubation at room temperature. Maximum enzyme activity was recorded on the fifth day of incubation, biomass after the seventh day, total protein and sugar released on the sixth day of incubation. Enzyme pretreatment of paper reduced 3.5 points in kappa number, 3.1 points increase in brightness and removal of chromophores and hydrophobic compounds. The FTIR and SEM analysis of enzyme-treated sample had shown modification in surface morphology and functional groups. These results clearly demonstrated that the xylanase produced by *A. niger* was effective as a pulp biobleaching agent which can be used on an industrial scale.

## Introduction

Considerable interest has been focused on the use of hydrolytic enzymes like xylanases that degrade xylan components in plant cell walls into simple sugars. Commercial applications of xylanases include pulp bleaching, food and animal feed industries, fuel, textile industries and in water management. They are required in bulk amounts and have significant application in paper and pulp industries as hydrolysis of xylan releases lignin from paper pulp and reduce usage of chemical bleaching agents. Throughout world pulp and paper mill industrial effluents contain toxic and harmful organic compounds as byproducts of pulping and bleaching processes. These effluents contain toxic heavy metals, lignin and its derivatives in addition to colour-imparting phenol and resinous compounds (Valls and Roncero 2009). Dark colour of unbleached pulp is due to the deposition of lignin and to remove colour one or more bleaching sequences like chlorine bleaching, oxidizing or reducing chemicals and alkaline extractions are needed (Khandeparkar and Bhosle 2007; Ziaie-shirkolaee et al. 2008). Due to use of these strong oxidants, chlorinated lignins and phenols are discharged into wastewaters. To substitute chlorine and to implement environmentally sound bleaching sequences by chlorine dioxide, hydrogen peroxide, oxygen, or ozone, among others can be used to get “totally chlorine-free” (TCF) bleaching. The drawback to introduce these environmentally sound technologies in pulp and paper industry is because it is difficult to attain high brightness degree as residual lignin and lignin-derived compounds that are more recalcitrant to degradation in TCF bleaching. To overcome these difficulties, enzymatic biobleaching using xylanases and laccases is an efficient alternative in many industrial applications. The ability of xylanases to facilitate the bleaching of kraft pulp was first reported in 1986 by the Finnish group led by Dr Lisa Viikari. Commercially supplied enzymes like cellulase and xylanase (Jeffries et al. 1994; Pala et al. 2004; Pathak et al. 2011) or lab-scale microbially produced enzymes reported by several researchers are applied to deink various types of papers (Gübitz et al. 1998; Vyas and Lachke 2003; Soni et al. 2010; Singh et al. 2012; Maity et al. 2012).

The application of enzymes like xylanases, cellulases or laccases in paper pulp bleaching is important as they reduce release of pollutants during bleaching and can also enhance the bleaching effect of chemical reagents by affording substantial savings (Valls and Roncero 2009). There are several reports on bioleaching using xylanases or fungal laccases either individually or in combination (Valls and Roncero 2009; Garg et al. 2011; Eugenio et al. 2010; Kapoor et al. 2007). Of late, scientists evinced interest in using filamentous fungi for production of xylanases and cellulases. Among the filamentous fungi, *Aspergillus* species are one of the most explored organisms. There are reports on xylanase production and application in pulp biobleaching process by many species of *Aspergillus* which include *Aspergillus niger* (Khonzue et al. 2011) *A. terricola marchal* and *A. ochraceus* (Michelin et al. 2010); *A. niger, A. niveus* and *A. ochraceus* (Betini et al. 2009); *A. niveus* RP05 and *A. fumigatus* RP04 (Peixoto-Nogueira et al. 2009); *A. nidulans* and *A. awamori* (Techapun et al. 2003); *A. fumigatus* (Savitha et al. 2007), and *A. caespitosus* (Sandrim et al. 2005). The results from the present study also agree with the existing reports and related results are discussed below. But studies regarding crude cellulase having xylanase activity for deinking and pulp biobleaching are very limited. In view of the industrial importance of xylanase, the present study is focused on evaluation of the extracellular xylanase produced by a potent fungal strain *A. niger* which is the first report of *A. niger* from Eastern Ghats and its ability in paper pulp biobleaching.

## Materials and methods

### Microorganisms and maintenance

The fungal strain *A. niger* used in this study was isolated from forest soil of Tirumala located in the eastern ghats of Andhra Pradesh, India, on potato dextrose agar medium. The stock cultures were maintained at 4 °C on slants of the same medium and deposited in our microbiology laboratory. Spore suspension (2 × 10^6^ count) was inoculated into a 250-ml Erlenmeyer flask containing 100 ml of MYG fermentation medium (glucose, 3 g; yeast extract, 0.8; K_2_HPO_4_, 0.4; MgSO_4_⋅7H_2_O, 0.2; pH, 7.0 and incubated at 28 ± 2 °C at 180 rpm for 7 days in a rotary shaking incubator.

### Screening

Screening of xylanase producing fungal strain was done in our laboratory using PDA medium with 1 % birchwood xylan. Five mm disc of the 4-day culture was placed the medium and incubated at 37 °C. After 3 days of incubation, the plates were stained with 1 % Congo Red and destained with 1 M NaCl. The best xylanase producer was selected for further studies based on the extent of formation of clear zone.

### Determination of xylanase activity

After incubation, the cultured broth was centrifuged (10,000 rpm) using a high-speed centrifuge for 15 min and the supernatant was used for enzyme assay. Xylanase activity was determined by the method of Bailey et al. (1992). Reaction mixture consisting 0.5 ml of culture filtrate with 0.5 ml of 1 % birchwood xylan [prepared in citrate buffer (0.05 M, pH 5.0)] was incubated for 15 min at 50 °C. After incubation the reaction was ceased by the addition of 1.5 ml of 3,5 dinitrosalicylic acid (Miller 1959) and heated for 10 min in boiling water bath. After cooling the reducing sugars liberated were measured spectrophotometrically at 540 nm. One unit of enzyme activity was defined as the amount of enzyme required to liberate 1 mol of reducing sugars per minute.

### Assay of cellulase

Estimation of cellulase activity was tested by Dinitrosalicylic acid (DNS) reagent (Miller 1959) which involves estimation of released reducing sugars from carboxy methyl cellulose (CMC) in 0.05 M phosphate buffer. Crude enzyme was added to 0.5 ml of 1 % CMC in 0.05 M phosphate buffer and incubated at 50 °C for 30 min. After incubation, the reaction was halted by the addition of 1.5 ml of DNS reagent and boiled at 100 °C in a water bath for 10 min. Released sugars were estimated by measuring absorbance at 540 nm. One unit of enzyme activity is expressed as the amount of enzyme, which is required to release 1 mol of glucose per minute under standard assay conditions.

### Characterization of paper pulp

Paper pulp was prepared by soaking printing paper waste in distilled water for 2 h, macerated in a domestic mixer and was oven-dried at 50 °C. Enzymatic bleaching of paper pulp was performed at a consistency of 3 % (w/v) and was mixed with crude enzyme (xylanase, 20–100 U/g of pulp) and incubated at 50 °C for 2 h. The treated pulp was filtered and the filtrate was used for biochemical analysis. Residual pulp was washed to prepare hand sheets by pressing between two stainless steel plates to form hand sheets and were dried at 50 °C for 3 h. A control was maintained without enzyme. The brightness of the pulp was determined according to the method of Jordan and Popson (1994). Chromophores, hydrophobic compounds of filtrate were measured at 237 and 465 nm and reducing sugars were determined by DNS method (Miller 1959).

### Scanning electron microscopic analysis

Morphological differences in pulp before and after the enzyme pretreatment were visualized by JEOL JSM-5600 scanning electron microscope. Images were taken at a magnification of 150×. Specimens to be coated were mounted on a conductive tape and coated with gold palladium using a JEOL-JFC-1200 fine coater and observed using a voltage of 25 kV.

### FTIR analysis

FTIR was recorded on Perkin-Elmer IR instrument to thoroughly understand the morphological changes in paper pulp. Functional groups before and after enzymatic treatment were characterized and spectra of both samples presented.

## Results and discussion

**Fig. 1:**
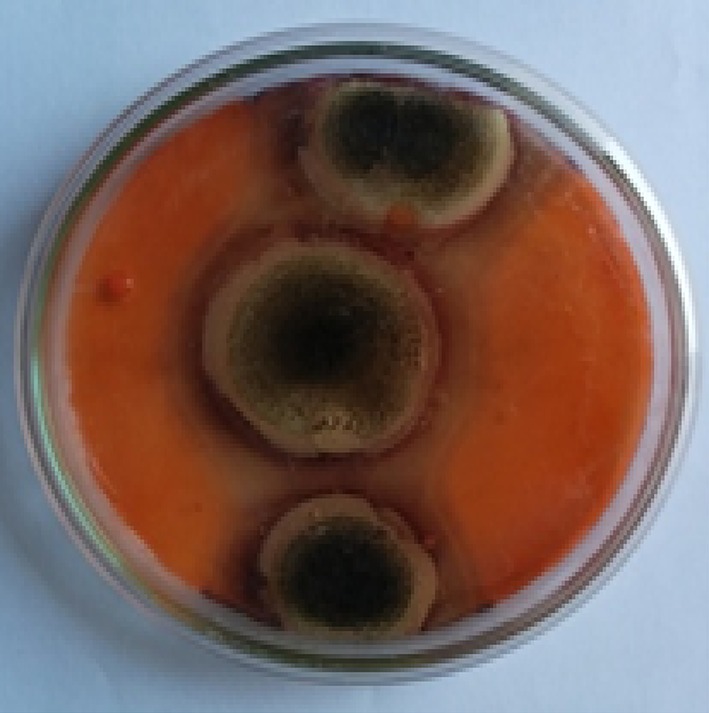
Isolation and screening of *A. niger* for production of xylanase

The isolates that had produced clear zone around colonies on MYG and CMC media after staining with Congo Red followed by destaining were recorded positive for xylanase and cellulase enzymes. Of them, one of the potential isolates (S5) was checked for its ability in quantitative secretion of hydrolytic enzymes and bioleaching of paper pulp. Preliminary microscopic examination of the isolate led to its identification as *Aspergillus* species and its screening is depicted in Fig. 1. The quantitative screening of the isolate and its enzyme activity is shown in Table 1. Enzyme production assays revealed that the isolate *A. niger* is both cellulolytic and xylanolytic. It was observed that maximum production of xylanase and cellulase occurred on the 5th day of incubation, but the present strain secreted higher xylanase (453.9 U/ml) when compared to cellulase (22.7 U/ml). Biomass, soluble sugar and protein secretion contents are shown in Table 2. Growth of fungal strain increased with incubation and reached maximum on the 7th day of incubation. Secreted total protein and sugar were found to be maximum after 6 days of incubation.

**Table 1:** Quantitative screening for xylanase production by *A. niger*

Day of incubation	Xylanase activity (U/mL)	Cellulase activity (U/ml)	Change in pH
2	12.6	3.5	3.1
3	64.8	10.3	3.7
4	121.7	17.5	4.2
5	453.9	22.7	4.9
6	397.3	15.2	5.3
7	276.3	9.4	5.5

**Table 2:** Biomass, total protein and total sugar content of *A. niger* at different days of incubation

Day of incubation	Biomass (mg/flask)	Total protein (µg/ml)	Total sugar (µg/ml)
2	240	176	262
3	478	386	312
4	651	493	597
5	824	674	768
6	946	896	924
7	1043	812	845

### Molecular characterization

**Fig. 2:**
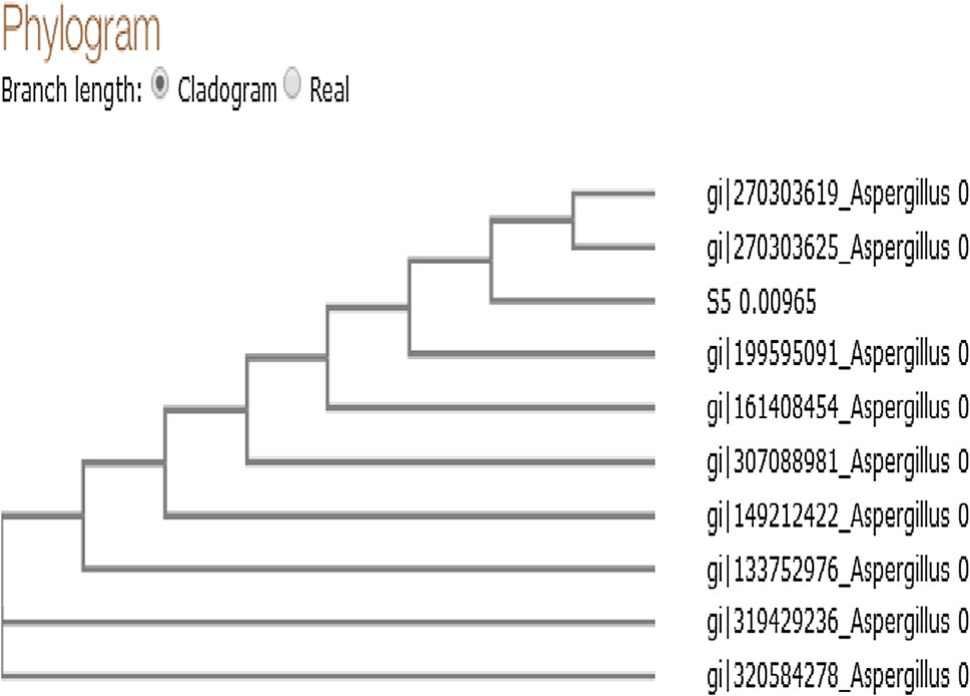
Phylogenetic tree of potential isolate constructed using NCBI

The 18S rRNA sequence was determined to investigate the relatedness of this strain to other species. Comparison of the sequence of the strain with the closest described species showed a 100 % similarity with *A. niger* and the strain was deposited in the NCBI nucleotide sequence databases with an accession number of KT727925. The phylogenetic tree was constructed as shown in Fig. 2.

### Enzymatic characterization of paper pulp

Xylanase and laccase enzymes may reduce hexenuronic acid (HexA) content of pulp by releasing xylans with xylanases, or direct oxidation with laccases or via yet unknown mechanism (Cadena et al. 2010). Hexenuronic acids that formed during alkaline cooking of pulp increase kappa number and reverse brightness and show adverse effects on pulp bleaching (Fillat et al. 2011). In support of this, the pulp bleaching potential of the crude xylanase from *A*. *niger* was evaluated by estimation of kappa number, brightness and reducing sugars. After enzyme treatment kappa number decreased and brightness of the pulp increased. Treatment after 1 h with 60 U/g dry pulp decreased 3.5 points in kappa number when compared to control. Kappa number is an indication of the lignin content or bleach ability of paper pulp. Further it has shown reduction in kappa number as well as 3.1 points increase in brightness after the enzyme treatment. Measurement of chromophores and hydrophobic compounds absorbance at 237 and 465 nm expected releasing from lignin and hemicelluloses (Table 3). Hemicellulases have also been proposed to cleave hemicellulose bonds near points of attachment between lignin and hemicellulose and it is possible that this leads to the improved solubilization of lignin.

**Table 3:** Properties of untreated and xylanase-treated pulp

Parameter	Untreated	Xylanase treated (60 U/g)
Kappa number	23.3	19.8
Brightness (ISO units)	38.7	41.8
Chromophoric compounds (*λ*_237_)	0.193	0.689
Hydrophobic compounds (*λ*_465_)	0.093	0.213
Reducing sugar (mg/g)	1.14	2.23

It was observed that with increase in time and increasing enzyme concentration there was 3.9 points decrease in the kappa number after 3 h of treatment and the brightness improved to 2.2, 2.8 and 3.1 points following treatment with 10, 20 and 40 U/g dry pulp, respectively, during 2 h of treatment (Guimarães et al. 2013). Using xylanase of *A. japonicus* (40 U/g dry pulp/2 h) the brightness of pulp improved 3.1 points, while the xylanase of *A. ochraceus* (35 U/g dry pulp/2 h) improved the brightness just 2.0 points (Betini et al. 2009). Free reducing sugar released after enzyme treatment from pulp was found to be 1.09 mg/g in this study. The effectiveness of enzyme pretreatment on pulp and release of reducing sugars was evaluated by other researchers like 33 mg of reducing sugar per gram of wheat straw pulp with 5 U/g, 25.78 mg/g of pulp after 3 h of incubation, 416 mg/g of pulp with purified xylanase of 150 U/g of pulp (Guimarães et al. 2013; Li et al. 2006; Bissoon et al. 2002). Even though release of reducing sugar is maximum in other reports this study limited in enzyme dose in bleaching process because enzyme concentration and loading is important in pulp bleaching as higher enzyme dose may damage to pulp fibres (Jurasek and Paice 1986).

**Fig. 3:**
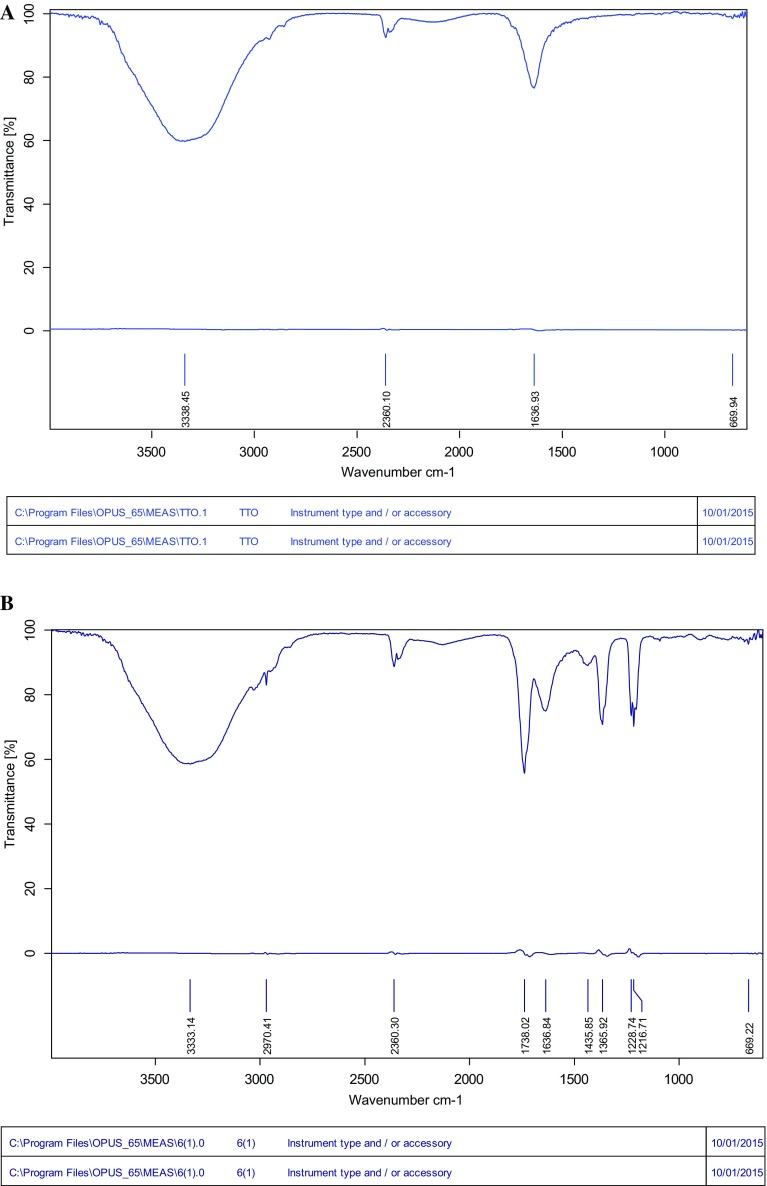
**a** Untreated paper pulp FTIR spectra. **b** Enzyme-treated paper pulp

**Fig. 4:**
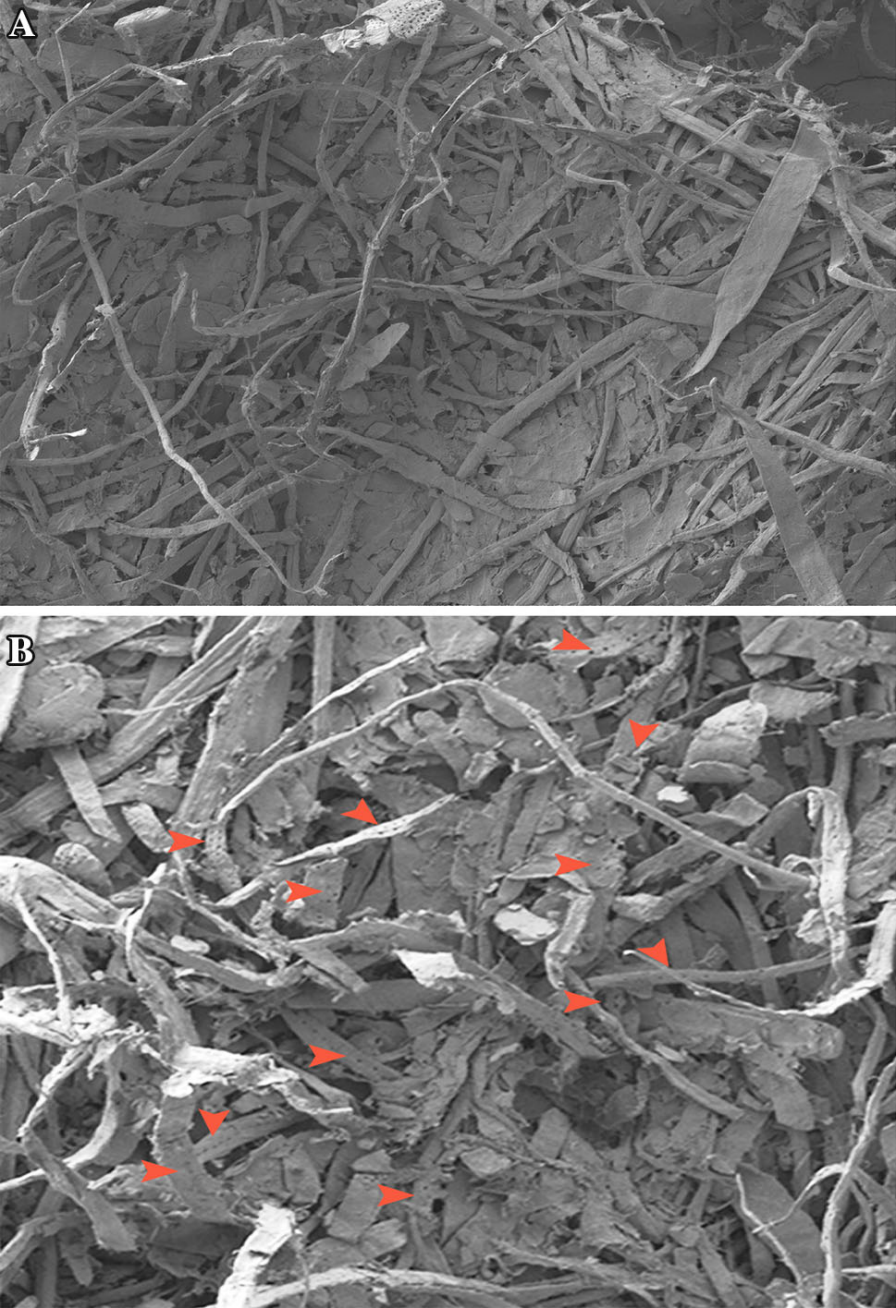
**a** Untreated paper pulp SEM image. **b** Enzyme-treated paper pulp

Reduction in kappa number, brightness increase and release of reducing sugars showed the potentiality of this fungal strain and its crude xylanase effect in paper and pulp industry. The FTIR measurements of paper pulp were carried out to identify the differences before and after enzyme pretreatment. The FTIR spectrum (Fig. 3a, b) showed peaks at different wavelengths corresponding to structural changes. The broad peak at 3000–3333 cm^−1^ indicated the O–H stretching while the band at 2360 cm^−1^ is due to –OH stretching of carboxylic acid in enzymatic treated pulp. The band at 1730 is due to C=O stretch of hemicelluloses and from 1636 to 1236; 1180 to 600 cm^−1^ is C–H and C–O bendings hemicellulose, respectively. Lignin and hemicellulose deformations were clear by observing above results in the treated pulp. A spectrum in FTIR is based on the infrared radiation absorption at frequencies that match those of the normal modes of vibration within the macromolecule. These absorption features are characteristics of the molecular configuration, sequencing and conformation. The intensity of an absorption band is related to the dipole moment change associated with the molecular vibration. The observed IR frequencies are given in Table 4. The characterization and interpretation of various functional groups are shown in Fig. 3a, b and Table 4. SEM images had also shown change in morphology of pulp fibres with breaking and appearance of pores in fibres (Fig. 4a, b).

**Table 4:** Characterization and interpretation of various functional groups

Untreated		Enzyme-treated	
Absorption ranges	Type of vibration	Absorption ranges	Type of vibration
Ranges (cm^−1^)		Ranges (cm^−1^)	
3333.45	–OH stretching of hydrogen-bonding	3333.14	–OH stretching of hydrogen-bonding
2925.80	=C–H stretch	2970.41	–
2360.10	OH asymmetrical stretching vibration in carboxylic acids	2360.30	OH asymmetrical stretching vibration in carboxylic acids
2138	–	1738.02	CH asymmetrical stretching vibration in CH3, CH2
1636.93	C=O stretch	1636.84	C=O stretch, amides, C=C stretching
		1435.85	C=H stretching
1312.60	–	1365.92	C=O stretch vibration in syringyl ring
1048.06	C–H	1228.74	C–O stretching
		1216.71	
1183.74	–	1183.74	–COO– (carboxylate ion) groups
669.94	N–O stretch	669.92	N–O stretch
647.76	–	647.76	C–O
616	–	616	C=O stretch

## Conclusions

Maximum yield of xylanase was produced by *A. niger* used in this study after 5 days of incubation when 1 % xylan was used as the substrate. Moreover, the strain has also exhibited ability to produce cellulase in low quantities. Crude enzyme dose had reduced kappa number and increased brightness of paper pulp. Change in functional groups identified by FTIR and surface morphological changes in fibres of pulp obtained by SEM results had confirmed that the present strain *A. niger* is effective in pulp biobleaching and can be explored on an industrial scale.
